# Association of modifiable risk factors with progression to dementia in relation to amyloid and tau pathology

**DOI:** 10.1186/s13195-024-01602-9

**Published:** 2024-10-26

**Authors:** Zsolt Huszár, Alina Solomon, Marie Anne Engh, Vanda Koszovácz, Tamás Terebessy, Zsolt Molnár, Péter Hegyi, András Horváth, Francesca Mangialasche, Miia Kivipelto, Gábor Csukly

**Affiliations:** 1 Centre for Translational Medicine, Semmelweis University, Üllői Út 26, Budapest, Hungary; 2 Department of Psychiatry and Psychotherapy, Semmelweis University, Balassa U. 6, Budapest, 1083 Hungary; 3 Institute of Clinical Medicine/Neurology, University of Eastern Finland, Kuopio, Finland; 4 Division of Clinical Geriatrics, Center for Alzheimer Research, Department of Neurobiology, Care Sciences and Society, Karolinska Institutet, Stockholm, Sweden; 5 Ageing Epidemiology Research Unit, School of Public Health, Imperial College London, London, UK; 6 Department of Anesthesiology and Intensive Therapy, Semmelweis University, Üllői 78/A, Budapest, Hungary; 7 Department of Anesthesiology and Intensive Therapy, Poznan University of Medical Sciences, 49 Przybyszewskiego St, Poznan, Poland; 8 Institute for Translational Medicine, Medical School, University of Pécs, Szigeti U. 12, Pécs, Hungary; 9 Institute of Pancreatic Diseases, Semmelweis University, Tömő 25-29, Budapest, Hungary; 10 Translational Pancreatology Research Group, Interdisciplinary Centre of Excellence for Research Development and Innovation, University of Szeged, 6728 Szeged, Hungary; 11 Neurocognitive Research Center, National Institute of Mental Health, Neurology, and Neurosurgery, Budapest, Hungary; 12 Department of Anatomy, Histology and Embryology, Semmelweis University, Budapest, Hungary; 13 Research Centre for Natural Sciences, Hungarian Research Network, Budapest, Hungary; 14 Institute of Public Health and Clinical Nutrition, University of Eastern Finland, Kuopio, Finland; 15 Theme Inflammation and Aging, Medical Unit Aging, Karolinska University Hospital, Stockholm, Sweden

**Keywords:** Modifiable risk factors, CAIDE, Depression, Smoking, Amyloid, Tau, MCI

## Abstract

**Background:**

Dementia preventive interventions targeting multiple modifiable risk factors are a promising approach. However, the impact of modifiable risk factors in the presence of beta-amyloid or phosphorylated-tau (p-tau) pathology is unclear.

**Methods:**

The objective of the study was to examine the role of modifiable risk factors (vascular factors, depression, and smoking) in the progression to mild cognitive impairment (MCI) or dementia among 434 cognitively unimpaired (CU) and 611 individuals with MCI from the Alzheimer's Disease Neuroimaging Initiative (ADNI) database. Vascular risk factors were summarized with the Cardiovascular Risk Factors, Aging, and Dementia (CAIDE) score, dichotomized into higher versus lower risk. Depression and smoking (yes/no) were categorised according to medical history or current symptoms. Analyses were stratified by beta-amyloid negative (A-) and positive (A +), p-tau negative (T-) and positive (T +), or beta-amyloid and p-tau negative (A-T-) and positive (A + T +) biomarker status. Cox proportional hazard models were adjusted for age, sex, education, baseline MMSE score, baseline hippocampal volume and ApoE4 carrier status.

**Results:**

Higher CAIDE score was associated with increased risk of progression to all-cause dementia in most MCI subgroups: adjusted hazard ratios (aHR) [95% CI] were 3.1 [1.43; 6.53] in the A- subgroup, 1.7 [1.20–2.27] in T + , 2.6 [1.06–6.59] in A-T-, and 1.6 [1.15–2.22] in the A + T + subgroup. Smoking (yes/no) was associated with increased dementia aHR in the A + MCI subgroup: 1.6 [1.07–2.34]. Depression increased dementia aHR in the T + MCI subgroup: 1.5 [1.06–2.02]. No significant associations were found in the CU biomarker subgroups.

**Conclusion:**

Addressing modifiable risk factors carries an important potential for reducing the risk of dementia even after the onset of Alzheimer's pathology. Knowledge of biomarker status can further optimize prevention strategies.

**Supplementary Information:**

The online version contains supplementary material available at 10.1186/s13195-024-01602-9.

## Background

Alzheimer’s disease (AD) and other forms of dementia are major causes of years lived with disability and represent a substantial long-term economic challenge for society. As the population ages, the consequences of dementia are anticipated to become even more severe [1]. Although there have been recent advances in anti-amyloid agents [2], current pharmacological therapeutic options have limited benefits. Addressing modifiable risk factors e.g. via lifestyle-based intervention programs in early risk and/or disease stages has been recommended for dementia risk reduction [3]. Major risk factors including e.g. smoking, depression, high blood pressure, and obesity, were estimated to account for about 40% of dementia cases [4, 5]. These risk factors have been linked to both AD and cerebrovascular damage [6–16].

To estimate an individual’s risk of developing dementia based on vascular factors, risk scores such as CAIDE (Cardiovascular Risk Factors, Aging, and Incidence of Dementia) have been developed [17]. The CAIDE score is based on age, education, sex, blood pressure, body mass index, total cholesterol, and physical activity. It provides a comprehensive and integrated assessment of an individual’s risk profile, allowing a more accurate estimate of the overall dementia risk, simplifying complex information into a single score, and making it more accessible to individuals and health professionals. From the above risk factors, obesity, high blood pressure, and hyperlipidemia are well known to increase the risk of vascular disease and, thus, the likelihood of cerebrovascular damage. They may also play a role in the development of AD [18, 19]. In the context of obesity and hyperlipidemia, adipokines and cholesterol have been described to modulate amyloid precursor protein degradation and thus beta-amyloid (Aβ) accumulation. Hypertension may also impair Aβ clearance, and may thus directly contribute to AD [20, 21].

The CAIDE dementia risk score has been previously tested in observational studies in relation to various cerebrospinal fluid (CSF) and neuroimaging markers, and post-mortem brain pathology [22], and higher scores correlate with signs of neurodegeneration such as reduced cortical thickness, increased medial temporal atrophy, white matter lesions, reduced brain perfusion, increased neuroinflammation, and changes in CSF Aβ and total tau [23–27]. It was also used to identify older at-risk individuals from the general population in the Finnish Geriatric Intervention study to prevent cognitive impairment and disability (FINGER). The FINGER trial showed cognitive and other related health benefits for a 2-year multidomain lifestyle intervention versus regular health advice [28]. In the Multidomain Alzheimer's Preventive Trial (MAPT), cognitive benefits from the multidomain intervention were shown in participants with a higher CAIDE score [29]. While a higher CAIDE score may reflect the potential for lifestyle-based dementia risk reduction in individuals without substantial impairment, its associations with dementia risk are less clear in populations with specific cognitive and neuropathological profiles.

The harmful effects of smoking on blood vessels, including in the brain, are well known [30, 31]. Smokers have an increased risk of dementia compared to those who have never smoked [14]. Moreover, there is evidence suggesting direct impact on AD development. Older smokers have reduced grey matter density in brain regions associated with the early stages of AD [32]. In vitro and animal studies have shown that cigarette smoke exposure consistently promotes amyloidogenic and tau abnormalities [15, 16]. Smoking is associated with cerebral oxidative stress, which promotes hyperphosphorylation of tau proteins and increases β-secretase cleavage of amyloid precursor protein involved in the production of Aβ oligomers and extracellular fibrillar Aβ aggregation [11].

Depression has been indicated as a risk factor for cognitive impairment in the context of vascular conditions as it is associated with adverse cerebrovascular effects, including increased risk of stroke and vascular pathological changes, which contribute to cognitive decline, and are also strongly associated with AD [6, 9, 33, 34]. Some studies have also reported that individuals with mild cognitive impairment (MCI) and pathological Aβ levels who have depressive symptoms progress more quickly to dementia than those without depressive symptoms [35–37].

The typical pathological changes in Aβ and tau proteins associated with Alzheimer’s disease appear decades before cognitive symptoms [38]. Detection of these protein changes in cognitively unimpaired (CU) or MCI individuals indicates a significant increase in the risk of cognitive decline [39–41]. While modifiable risk factors may provide room for dementia risk reduction, associations of the CAIDE risk score and additional risk factors such as depression and smoking with clinical progression in populations with more specific cognitive-neuropathological profiles is not fully clear. In the present study, we aimed to examine the role of defined modifiable risk factors, namely the CAIDE score, depression, and smoking, in the progression to MCI or all-cause dementia among biomarker-homogeneous (in terms of Aβ and p-tau) CU and MCI subgroups. This was accomplished by performing a comparative analysis of progression data between participants who were either positive or negative for these modifiable risk factors within each subgroup, classified according to Aβ, p-tau, and both Aβ and p-tau pathology.

## Methods

### Study population

Data from 1045 (611 with MCI and 434 cognitively unimpaired) participants in the Alzheimer’s Disease Neuroimaging Initiative (ADNI) were used. ADNI is a publicly available (https://adni.loni.usc.edu/) follow-up study cohort at more than 60 clinical sites in the US and Canada that uses a variety of biomarkers, neuroimaging, and clinical assessments to study Alzheimer's disease and dementia. Enrolled participants were categorised into CU, MCI and all-cause dementia groups using the Clinical Dementia Rating (CDR) score (CDR = 0 for CU, CDR = 0.5 for MCI and > 0.5 for dementia) and education level adjusted MMSE and Wechsler Logical Memory II subscale tests to aid in the diagnostic process. Participants were aged between 55 and 90 years and underwent a comprehensive medical examination. Individuals with severe neurological or psychiatric disorders and systemic diseases affecting cognition were excluded from the study. Full details of the enrolment process are available at https://adni.loni.usc.edu/help-faqs/adni-documentation/. The date of the ADNI database download was May 05, 2022, with data captured from 2005 onwards. CU and MCI were assessed using participant-level follow-up data (see Supplementary Appendix 1 for detailed ADNI data management) [42].

CU and MCI subgroups were classified according to Aβ, p-tau, or both Aβ and p-tau pathology. Analyses of the various dementia risk factors for the CU group were performed on data from 434 participants when considering Aβ pathology alone, 331 participants when considering p-tau pathology alone, and 219 participants when considering both Aβ and p-tau pathology. Analyses of the MCI group for Aβ pathology alone were based on data from 611 participants, for p-tau pathology alone on 551 participants, and on 417 participants when both pathologies were considered together. The median follow-up for both CU and MCI participants was four years. Detailed baseline data and progression to MCI or all-cause dementia during follow-up are shown in Tables 1 and 2.

**Table 1:** Baseline information - Cognitively Unimpaired (CU)

**Amyloid**	*n*	**All** (*n* = 434)	**Amyloid positive** (*n* = 152)	**Amyloid negative** (*n* = 282)	**statistics**
Age: mean (SD) years	434	73.3 ( 6.2)	74.7 ( 5.9)	72.5 ( 6.2)	t = -4.3,df = 346.0,*p* = < .0001 *
Baseline MMSE	434	29.1 ( 1.2)	29.0 ( 1.1)	29.1 ( 1.2)	t = 0.1, df = 363.2, *p* = 0.9501
ApoE4 carrier status	434	124 ( 28.6%)	70 ( 46.1%)	54 ( 19.1%)	ChiSq = 35.0,df = 1.0,*p* = < .0001 *
Baseline Hippocampus Volume (mm3)	434	7483 ( 864)	7336 ( 860)	7562 ( 857)	t = 2.6, df = 307.7, *p* = 0.0089
Female gender: *n* (%)	434	240 ( 55.3%)	94 ( 61.8%)	146 ( 51.8%)	ChiSq = 4.1, df = 1.0, *p* = 0.0441
Higher CAIDE score: *n* (%)	428	131 ( 30.6%)	48 ( 31.8%)	83 ( 30.0%)	ChiSq = 0.2, df = 1.0, *p* = 0.6956
CAIDE Total Score	428	5.8 ( 1.5)	5.8 ( 1.5)	5.8 ( 1.4)	t = -0.3, df = 318.7, *p* = 0.7691
Depression as risk: *n* (%)	434	74 ( 17.1%)	28 ( 18.4%)	46 ( 16.3%)	ChiSq = 0.3, df = 1.0, *p* = 0.5773
Smokers: *n* (%)	356	40 ( 11.2%)	17 ( 13.6%)	23 ( 10.0%)	ChiSq = 1.1, df = 1.0, *p* = 0.2988
Follow-up time: median(IQR)	434	48 ( 24- 96)	48 ( 24- 90)	60 ( 24- 96)	ChiSq = 3.2, df = 1.0, *p* = 0.0724
Progression to MCI or dementia: *n* (%)	434	83 ( 19.1%)	38 ( 25.0%)	45 ( 16.0%)	ChiSq = 5.2, df = 1.0, *p* = 0.0223
**p-tau181**	***n***	**All (*n*** **= 331)**	**p-tau181 positive (*n*** **= 114)**	**p-tau181 negative (*n*** **= 217)**	**statistics**
Age: mean (SD) years	331	74.0 ( 5.8)	75.7 ( 6.1)	73.1 ( 5.4)	t = -3.9,df = 220.4,*p* = 0.0001 *
Baseline MMSE	331	29.0 ( 1.2)	29.0 ( 1.2)	29.0 ( 1.1)	t = 0.1, df = 227.2, *p* = 0.8962
ApoE4 carrier status	331	88 ( 26.6%)	39 ( 34.2%)	49 ( 22.6%)	ChiSq = 5.2, df = 1.0, *p* = 0.0229
Baseline Hippocampus Volume (mm3)	331	7452 ( 856)	7313 ( 881)	7525 ( 836)	t = 2.1, df = 219.4, *p* = 0.0354
Female gender: *n* (%)	331	171 ( 51.7%)	59 ( 51.8%)	112 ( 51.6%)	ChiSq = 0.0, df = 1.0, *p* = 0.9805
Higher CAIDE score: *n* (%)	327	105 ( 32.1%)	41 ( 36.6%)	64 ( 29.8%)	ChiSq = 1.6, df = 1.0, *p* = 0.2087
CAIDE Total Score	327	5.8 ( 1.5)	5.9 ( 1.5)	5.8 ( 1.5)	t = -0.7, df = 241.3, *p* = 0.5003
Depression as risk: *n* (%)	331	68 ( 20.5%)	23 ( 20.2%)	45 ( 20.7%)	ChiSq = 0.0, df = 1.0, *p* = 0.9043
Smokers: *n* (%)	331	37 ( 11.2%)	10 ( 8.8%)	27 ( 12.4%)	ChiSq = 1.0, df = 1.0, *p* = 0.3139
Follow-up time: median(IQR)	331	72 ( 36–102)	66 ( 36- 96)	72 ( 36–102)	ChiSq = 0.1, df = 1.0, *p* = 0.7322
Progression to MCI or dementia: *n* (%)	331	72 ( 21.8%)	39 ( 34.2%)	33 ( 15.2%)	ChiSq = 15.9,df = 1.0, *p* = < .0001 *
**Amyloid and p-tau181**	***n***	**All (*n*** **= 219)**	**Amyloid and p-tau181 positive (*n*** **= 60)**	**Amyloid and** **p-tau181 negative (*n*** **= 159)**	**statistics**
Age: mean (SD) years	219	73.7 ( 5.6)	76.4 ( 5.2)	72.6 ( 5.5)	t = -5.1,df = 119.5,*p* = < .0001 *
Baseline MMSE	219	29.1 ( 1.2)	29.1 ( 1.1)	29.1 ( 1.2)	t = -0.4, df = 122.3, *p* = 0.6813
ApoE4 carrier status	219	56 ( 25.6%)	28 ( 46.7%)	28 ( 17.6%)	ChiSq = 19.3,df = 1.0,*p* = < .0001 *
Baseline Hippocampus Volume (mm3)	219	7492 ( 800)	7245 ( 817)	7585 ( 776)	t = 2.8, df = 101.7, *p* = 0.0064
Female gender: *n* (%)	219	109 ( 49.8%)	33 ( 55.0%)	76 ( 47.8%)	ChiSq = 0.9, df = 1.0, *p* = 0.3418
Higher CAIDE score: *n* (%)	216	70 ( 32.4%)	23 ( 39.0%)	47 ( 29.9%)	ChiSq = 1.6, df = 1.0, *p* = 0.2056
CAIDE Total Score	216	5.8 ( 1.5)	5.9 ( 1.5)	5.8 ( 1.5)	t = -0.7, df = 108.2, *p* = 0.5080
Depression as risk: *n* (%)	219	42 ( 19.2%)	12 ( 20.0%)	30 ( 18.9%)	ChiSq = 0.0, df = 1.0, *p* = 0.8495
Smokers: *n* (%)	219	22 ( 10.0%)	6 ( 10.0%)	16 ( 10.1%)	ChiSq = 0.0, df = 1.0, *p* = 0.9890
Follow-up time: median(IQR)	219	72 ( 36- 96)	54 ( 36- 90)	72 ( 36–102)	ChiSq = 4.7, df = 1.0, *p* = 0.0305
Progression to MCI or dementia: *n*(%)	219	46 ( 21.0%)	23 ( 38.3%)	23 ( 14.5%)	ChiSq = 15.0,df = 1.0,*p* = 0.0001 *

*CU* Cognitively Unimpaired, *MCI* Mild Cognitive Impairment, *MMSE* Mini-Mental State Examination, *ApoE4* Apolipoprotein E epsilon 4 carriers, *CAIDE* Cardiovascular Risk Factors, Aging, and Dementia, *p-tau181* Phosphorylated tau 181, *CSF* Cerebrospinal Fluid, *n* Number of participants, *SD* Standard Deviation, *IQR* Interquartile Range, *ChiSq* Chi-Square Test, *df* Degrees of Freedom, *p* p-value

*p-values indicate significant differences between biomarker positives and negatives (after correction for multiple comparisons *p* < 0.05/11, where 11 is the number of parameters compared), and are based on T-tests or Wilcoxon tests (follow-up time) in case of continuous variables and Chi-Square tests in case of categorical variables

**Table 2:** Baseline information - Mild Cognitive Impairment (MCI)

**Amyloid**	*n*	**All **(*n* = 611)	**Amyloid positive** (*n* = 377)	**Amyloid negative** (*n* = 234)	**statistics**
Age: mean (SD) years	610	72.5 ( 7.4)	73.5 ( 6.8)	70.9 ( 8.1)	t = -4.0,df = 481.4,*p* = < .0001 *
Baseline MMSE	611	27.8 ( 1.8)	27.4 ( 1.8)	28.4 ( 1.5)	t = 8.2,df = 630.7,*p* = < .0001 *
ApoE4 carrier status	611	300 ( 49.1%)	246 ( 65.3%)	54 ( 23.1%)	ChiSq = 102.8,df = 1.0,*p* = < .0001 *
Baseline Hippocampus Volume (mm3)	611	6865 (1133)	6631 (1064)	7242 (1142)	t = 6.6,df = 474.7,*p* = < .0001 *
Female gender: *n* (%)	611	255 ( 41.7%)	156 ( 41.4%)	99 ( 42.3%)	ChiSq = 0.1, df = 1.0, *p* = 0.8210
Higher CAIDE score: *n* (%)	606	223 ( 36.8%)	141 ( 37.8%)	82 ( 35.2%)	ChiSq = 0.4, df = 1.0, *p* = 0.5172
CAIDE Total Score	606	5.9 ( 1.4)	5.8 ( 1.4)	5.9 ( 1.5)	t = 0.8, df = 543.7, *p* = 0.4211
Depression as risk: *n* (%)	611	192 ( 31.4%)	110 ( 29.2%)	82 ( 35.0%)	ChiSq = 2.3, df = 1.0, *p* = 0.1290
Smokers: *n* (%)	576	70 ( 12.2%)	48 ( 13.5%)	22 ( 10.0%)	ChiSq = 1.5, df = 1.0, *p* = 0.2138
Follow-up time: median(IQR)	611	48 ( 30- 78)	48 ( 24- 60)	48 ( 36- 96)	ChiSq = 17.8,df = 1.0,*p* = < .0001 *
Progression to dementia: n(%)	611	221 ( 36.2%)	195 ( 51.7%)	26 ( 11.1%)	ChiSq = 103.2,df = 1.0,*p* = < .0001 *
**p-tau181**	***n***	**All (*n*** **= 551)**	**p-tau181 positive (*n*** **= 305)**	**p-tau181 negative (*n*** **= 246)**	**statistics**
Age: mean (SD) years	551	72.4 ( 7.5)	73.4 ( 7.4)	71.1 ( 7.4)	t = -3.4,df = 563.7,*p* = 0.0007 *
Baseline MMSE	551	27.7 ( 1.8)	27.4 ( 1.8)	28.1 ( 1.7)	t = 5.2,df = 578.7,*p* = < .0001 *
ApoE4 carrier status	551	273 ( 49.5%)	192 ( 63.0%)	81 ( 32.9%)	ChiSq = 49.1,df = 1.0,*p* = < .0001 *
Baseline Hippocampus Volume (mm3)	551	6820 (1150)	6582 (1077)	7116 (1171)	t = 5.5,df = 504.2,*p* = < .0001 *
Female gender: n (%)	551	230 ( 41.7%)	132 ( 43.3%)	98 ( 39.8%)	ChiSq = 0.7, df = 1.0, *p* = 0.4155
Higher CAIDE score: n (%)	548	196 ( 35.8%)	102 ( 33.7%)	94 ( 38.4%)	ChiSq = 1.3, df = 1.0, *p* = 0.2534
CAIDE Total Score	548	5.8 ( 1.4)	5.7 ( 1.3)	6.0 ( 1.5)	t = 2.2, df = 522.8, *p* = 0.0309
Depression as risk: n (%)	551	183 ( 33.2%)	90 ( 29.5%)	93 ( 37.8%)	ChiSq = 4.2, df = 1.0, *p* = 0.0398
Smokers: *n* (%)	551	68 ( 12.3%)	41 ( 13.4%)	27 ( 11.0%)	ChiSq = 0.8, df = 1.0, *p* = 0.3814
Follow-up time: median(IQR)	551	48 ( 36- 84)	48 ( 36- 66)	48 ( 36- 90)	ChiSq = 12.6,df = 1.0, *p* = 0.0004 *
Progression to dementia: *n* (%)	551	213 ( 38.7%)	163 ( 53.4%)	50 ( 20.3%)	ChiSq = 63.0,df = 1.0,*p* = < .0001 *
**Amyloid and p-tau181**	***n***	**All (*n*** **= 418)**	**Amyloid and** **p-tau181 positive (*n*** **= 257)**	**Amyloid and** **p-tau181 negative (*n*** **= 160)**	**statistics**
Age: mean (SD) years	417	72.1 ( 7.6)	73.4 ( 7.1)	69.9 ( 7.8)	t = -4.4,df = 343.3, *p* = < .0001 *
Baseline MMSE	417	27.7 ( 1.8)	27.3 ( 1.8)	28.4 ( 1.5)	t = 7.3,df = 419.3, *p* = < .0001 *
ApoE4 carrier status	417	213 ( 51.1%)	177 ( 68.9%)	36 ( 22.5%)	ChiSq = 84.9,df = 1.0, *p* = < .0001 *
Baseline Hippocampus Volume (mm3)	417	6763 (1142)	6477 (1026)	7221 (1174)	t = 6.6,df = 303.4, *p* = < .0001 *
Female gender: *n* (%)	417	184 ( 44.1%)	113 ( 44.0%)	71 ( 44.4%)	ChiSq = 0.0, df = 1.0, *p* = 0.9353
Higher CAIDE score: *n* (%)	414	144 ( 34.8%)	88 ( 34.5%)	56 ( 35.2%)	ChiSq = 0.0, df = 1.0, *p* = 0.8827
CAIDE Total Score	414	5.8 ( 1.4)	5.7 ( 1.3)	5.9 ( 1.6)	t = 1.3, df = 332.2, *p* = 0.2014
Depression as risk: *n* (%)	417	144 ( 34.5%)	78 ( 30.4%)	66 ( 41.3%)	ChiSq = 5.2, df = 1.0, *p* = 0.0228
Smokers: *n* (%)	417	45 ( 10.8%)	33 ( 12.8%)	12 ( 7.5%)	ChiSq = 2.9, df = 1.0, *p* = 0.0874
Follow-up time: median(IQR):	417	48 ( 36- 78)	48 ( 36- 60)	60 ( 36- 96)	ChiSq = 20.7,df = 1.0, *p* = < .0001 *
Progression to dementia: *n* (%)	417	178 ( 42.7%)	158 ( 61.5%)	20 ( 12.5%)	ChiSq = 96.7,df = 1.0, *p* = < .0001 *

*CU* Cognitively Unimpaired, *MCI* Mild Cognitive Impairment, *MMSE* Mini-Mental State Examination, *ApoE4* Apolipoprotein E epsilon 4 carriers, *CAIDE* Cardiovascular Risk Factors, Aging, and Dementia, *p-tau181* Phosphorylated tau 181, *CSF* Cerebrospinal Fluid, *n* Number of participants, *SD* Standard Deviation, *IQR* Interquartile Range, *ChiSq* Chi-Square Test, *df* Degrees of Freedom, *p* p-value

^*^p-values indicate significant differences between biomarker positives and negatives (after correction for multiple comparisons *p* < 0.05/11, where 11 is the number of parameters compared), and are based on T-tests or Wilcoxon tests (follow-up time) in case of continuous variables and Chi-Square tests in case of categorical variables

### Risk factors

The examined risk factors, such as depression, smoking, high blood pressure, obesity, and hyperlipidemia, were treated as dichotomous variables, and participants were categorised as having vs not having a risk factor according to their medical history. The CAIDE score was calculated based on age, sex, education, hypertension (Systolic Blood Pressure > 140 mm Hg), obesity (body mass index (BMI) > 30 kg/m^2^) and hyperlipidemia (total cholesterol > = 6.5 mmol/L) as previously described in detail (Supplementary eTable 1) [17]. All risk factors were measured at baseline, which was the starting (zero) point of the survival analyses. Physical activity could not be included in the CAIDE calculation because data were unavailable in the ADNI database. Based on the median CAIDE score and the cut-off previously used in the FINGER study [28], we used six points as a cut-off for high dementia risk.

Assignment to the smoking group was based on the participants' medical records. Similary, based on a history of depression documented in medical records, or baseline depressive symptoms, participants were divided into depression and no depression groups. Depressive symptoms were assessed using the Neuropsychiatric Inventory-Questionnaire (NPI-Q) in ADNI 1 or the Neuropsychiatric Inventory (NPI) in ADNI GO, ADNI 2, and ADNI 3 [43–45]. Following criteria established in previous studies for the CU and MCI populations, the cut-off point for categorizing depression was a severity score of ≥ 2 on the NPI-Q [46, 47] or a severity × frequency score of ≥ 4 on the NPI [48, 49].

### Aβ and p-tau status

We used the ^18^F-Florbetapir (AV45) PET data as the default Aβ measurement where available. Florbetapir standardized uptake value ratio (SUVR) was created by averaging the four cortical regions and dividing them by the cerebellum as a reference. According to the ADNI recommendation, we applied the SUVR cut-off of 1.11 and used the whole cerebellum region as a reference [50]. In a previous study [51] Florbetapir positivity defined using the same cut-off was shown to be strongly correlated with post-mortem autopsy results. If PET data were unavailable, we used Aβ1-42 CSF measurements (Roche Elecsys) to maximize the analysis sample size. As previously indicated [52], we applied a cut-off of 977 pg/ml for Aβ1-42 measurements since this cut-off value showed the highest agreement with amyloid PET results (overall percent agreement 87%, 95% CI 84.2–89.5). Participants were defined as p-tau positive by CSF p-tau181 levels (INNO-BIA AlzBio3) above 23 pg/ml, a cut-off shown in a previous study on autopsy-based AD cases to have the best classification power [53].

The rationale for analyzing data on Aβ status separately was twofold. First, p-tau status was available for a smaller number of participants, thus focusing only on Aβ increased the statistical power. Second Aβ captures a broader risk group who may not (yet) have tau pathology. However, abnormal p-tau alongside Aβ indicates a more severe condition. Therefore, we studied A + T + /A-T- subgroups as well. We also analyzed groups subdivided by CSF p-tau181 pathology alone (T + /T-), reflecting the 2024 classification [54], which indicates that *p*-tau181 becomes abnormal alongside amyloid PET, but before tau PET.

### Statistical analysis

The CU and MCI groups were divided into Aβ positive and negative (A + , A-), p-tau positive and negative (T + , T-) and Aβ and p-tau positive and negative (A + T + , A-T-) subgroups. Baseline characteristics of CU and MCI participants were compared between each biomarker positive and negative subgroup using t-test, Wilcoxon or Chi-square tests as appropriate. The associations of CAIDE score, depression, and smoking with progression to MCI and/or dementia were investigated in analyses stratified by cognitive and pathology status: CU A + /A-, CU T + /T-, CU A + T + /A-T-, MCI A + /A-, MCI T + /T-, MCI A + T + /A-T-.

We calculated the (adjusted) Hazard Ratios (HR) with their confidence interval (CI) from a Cox Proportional Hazard Model (PROC PHREG in SAS 9.4). Progression to dementia in the MCI group or progression to dementia and MCI combined (in the CU group) were the dependent (predicted) variables in separate models, while Aβ and p-tau positivity served as predictor variables together with modifiable risk factors such as CAIDE score, smoking, and depression. Cox regression (Cox) analyses of smoking and depression included age, sex, education, baseline MMSE score, baseline hippocampal volume and ApoE4 carrier status as covariates. Cox regression analyses of CAIDE score included age, baseline MMSE score, and baseline hippocampal volume and ApoE4 carrier status as covariates (sex and education were already included in the CAIDE score). In order to test the proportional hazard assumption we repeated all Cox regressions by including the interaction of time and risk factors as covariates. Since the interaction of time and risk factors were non-significant in all Cox regressions (all *p* values > 0.1) we can conclude that there is no evidence of the time dependency of the hazard ratios, i.e. the proportional hazard assumption were met in all cases. Death was included as a competing risk in the Cox regressions. All reported Hazard Ratios from Cox regressions are adjusted ones (aHR). Where a subgroup included < 20 participants, the survival analysis was not performed due to a high risk of bias.

The methodology described above was not applied to the CU and MCI A + T- and A-T + subgroups because of the high risk of bias due to the small sample size (< 20).

### Sensitivity analyses

The Kaplan–Meier survival analyses were also performed for all biomarker groups and risk factors. The survival plots are included in the figures; therefore, the adjusted curves from the Cox regressions and the survival plots are easily comparable. In the results section, we also present the statistics (log-rank test and corresponding *p* values) from the Kaplan–Meier (KM) analyses.

Furthermore, we performed the Cox regression analysis with the CAIDE total score as a continuous variable and with seven as alternate cut-off value for the CAIDE score. Finally, we analyzed the effect of CAIDE as risk factor in the MCI sample regardless of biomarker status.

## Results

Based on the analysis of 434 CU and 611 MCI participants, baseline characteristics did not differ significantly between the A-/A + , T-/T + , and A-T-/A + T + subgroups for either CU or MCI participants according to the percentage of participants with a higher CAIDE score, depression, and smoking. There were significant differences in age, ApoE4 carrier status, MMSE score, hippocampal volume and progression rate between the biomarker-negative and positive subgroups (Table 1 and 2).

A total of 103 CU and 60 MCI participants lacked p-tau data, with only data on their Aβ status available for analysis. The number of CU participants in each biomarker subgroup was 277 (A-), 151 (A +), 217 (T-), 114 (T +), 58 (A + T-), 53 (A-T +), 157 (A-T-), and 59 (A + T +). MCI participants included 234 (A-), 377 (A +), 246 (T-), 305 (T +), 86 (A + T-), 48 (A-T +), 160 (A-T-), and 257 (A + T +).

### Higher CAIDE score and progression to MCI and/or dementia

Among CU participants with higher CAIDE scores, compared to those with lower scores, the risk of progression to MCI or dementia was not significantly increased in either the biomarker-negative or biomarker-positive subgroups (Table 3, Supplementary eFigure 1). The KM analyses did not show a statistically significant difference between any CU/CAIDE risk groups (all p values > 0.1).

**Table 3:** The effect of modifiable risk factors on progression to MCI and/or dementia

Effect	**CU**				**MCI**			
	aHR (95% CI)		aHR (95% CI)		aHR (95% CI)		aHR (95% CI)	
**Higher CAIDE score**	A-	1.6 (0.89; 2.93)	A +	1.0 (0.49; 1.92)	A-	**3.1 (1.43; 6.53)**	A +	1.3 (0.98; 1.75)
	T-	1.1 (0.54; 2.40)	T +	1.0 (0.55; 2.01)	T-	1.6 (0.94; 2.83)	T +	**1.7 (1.20; 2.27)**
	A-T-	1.9 (0.80; 4.25)	A + T +	0.9 (0.39; 2.15)	A-T-	**2.6 (1.06; 6.59)**	A + T +	**1.6 (1.15; 2.22)**
**Smoking**	A-	n.e.	A +	n.e.	A-	1.3 (0.39; 4.23)	A +	**1.6 (1.07; 2.34)**
	T-	n.e.	T +	n.e.	T-	1.8 (0.89; 3.78)	T +	1.5 (0.99; 2.31)
	A-T-	n.e.	A + T +	n.e.	A-T-	n.e.	A + T +	n.e.
**Depression**	A-	1.6 (0.81; 3.36)	A +	1.0 (0.42; 2.60)	A-	1.0 (0.48; 2.19)	A +	1.2 (0.86; 1.57)
	T-	1.2 (0.44; 3.19)	T +	1.1 (0.48; 2.45)	T-	0.6 (0.30; 1.05)	T +	**1.5 (1.06; 2.02)**
	A-T-	n.e.	A + T +	n.e.	A-T-	0.6 (0.22; 1.49)	A + T +	1.3 (0.94; 1.84)

Bold numbers indicate a significant increase

*CU* Cognitively Unimpaired, *MCI* Mild Cognitive Impairment, *aHR* adjusted Hazard Ratio, *95% CI* 95% Confidence Interval, *A-* beta-amyloid negative, *A* + beta-amyloid positive, *T-* p-tau negative, *T* + p-tau positive, *n.e.* not estimated (due to small number of cases)

**Fig. 1:**
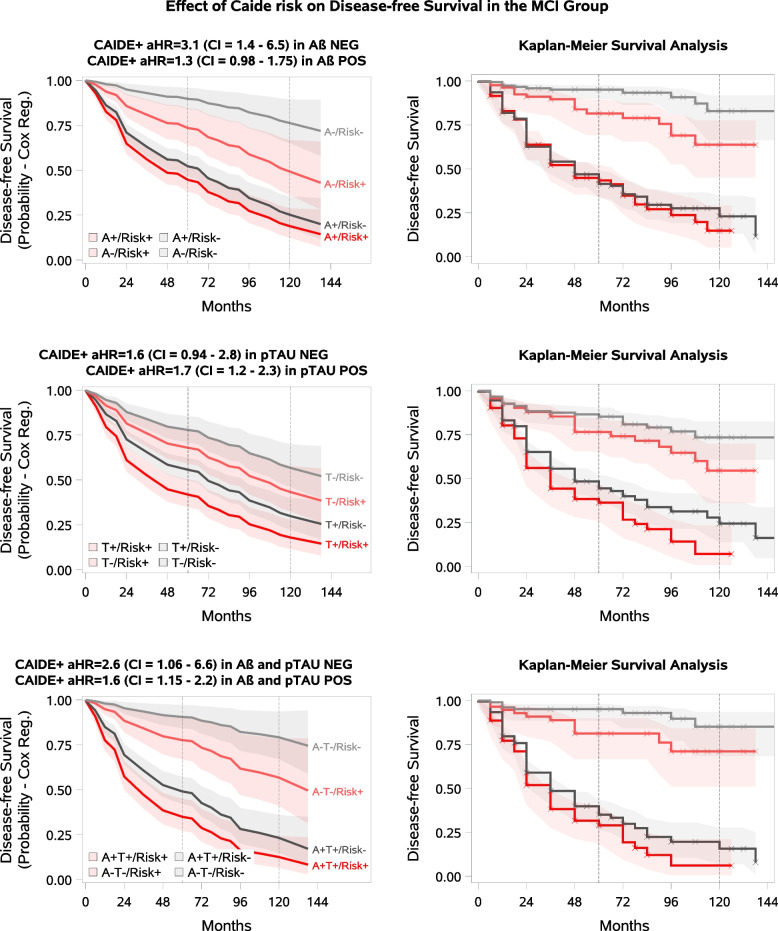
**CAIDE Score and Dementia Progression in MCI by beta-amyloid/p-tau Status.** The pale lines in the figure represent the biomarker-negative group, the solid lines represent the biomarker-positive group, the red lines represent the modifiable risk factor-positive group, and the grey lines represent the modifiable risk factor-negative group. The shaded areas represent the confidence intervals. Disease-free survival means no progression to dementia. **A** CAIDE as a modifiable risk factor in MCI A-/A + participants. **B** CAIDE as a modifiable risk factor in MCI T-/T + participants. **C** CAIDE as a modifiable risk factor in MCI A-T-/A + T + participants

In the MCI population (Table 3, Fig. 1), the risk of progression to dementia was significantly increased among A- MCI participants with higher compared to lower CAIDE scores (Cox aHR = 3.1, 95% CI 1.43–6.53, KM log-rank chi-square (ChiSq) = 8.1, *p* = 0.004), while in the A + MCI subgroup a statistical trend-level association was observed (Cox aHR = 1.3, 95% CI 0.98–1.7, KM log-rank ChiSq = 0.16, *p* = 0.7). In the T + subgroup, higher CAIDE score was related to higher dementia risk compared with lower CAIDE score (Cox aHR = 1.7 95%CI 1.20–2.27, KM log-rank ChiSq = 5.0, *p* = 0.03), with a similar trend in the T- subgroup (Cox aHR = 1.6, 95%CI 0.94–2.83, KM log-rank ChiSq = 2.8, *p* = 0.096). Higher CAIDE score was significantly associated with an increased progression risk among both the A-T- (Cox aHR = 2.6, 95%CI 1.06–6.59, KM log-rank ChiSq = 4.7, *p* = 0.03) and A + T + (Cox aHR = 1.6, 95%CI 1.15–2.22, KM log-rank ChiSq = 2.6, *p* = 0.1) MCI subgroups.

### Sensitivity analysis for CAIDE score and risk for progression in the MCI group

According to the literature, cut-offs higher than six are acceptable [55]. We conducted the sensitivity analysis with the cut-off score of seven and also the CAIDE total score as a continuous variable in the MCI group. With seven as cutoff, none of the aHRs remained significant, while for CAIDE as a continuous variable, one point increase in the total score was associated with an increased risk in the A- (Cox aHR = 1.4, 95%CI 1.1–1.8), A-T- (Cox aHR = 1.4, 95%CI 1.01–1.9), A + T + (Cox aHR = 1.1, 95%CI 1.01–1.3), and T + groups (Cox aHR = 1.1, 95%CI 1.01–1.3). In the whole MCI sample, regardless of the biomarker status, higher CAIDE scores were associated with an increased risk of preogression (Cox aHR = 1.5, 95CI 1.1–1.9).

### Smoking and progression to dementia

**Fig. 2:**
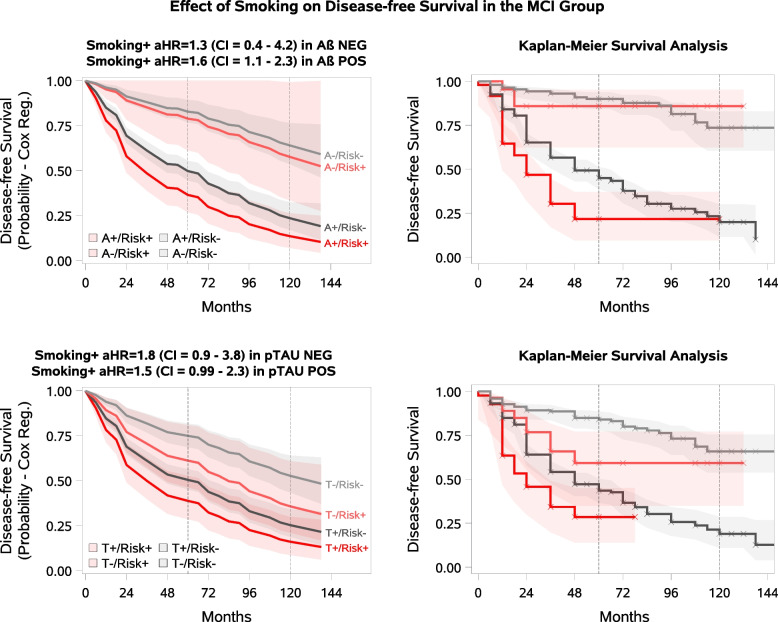
**Smoking and Dementia Progression in MCI by beta-amyloid /p-tau Status.** The pale lines in the figure represent the biomarker-negative group, the solid lines represent the biomarker-positive group, the red lines represent the modifiable risk factor-positive group, and the grey lines represent the modifiable risk factor-negative group. The shaded areas represent the confidence intervals. Disease-free survival means no progression to dementia. **A** Smoking as a modifiable risk factor in MCI A-/A + participants. **B** Smoking as a modifiable risk factor in MCI T-/T + participants

In the MCI population, the risk of progression to dementia was significantly increased in smokers compared to non-smokers in the A + (Cox aHR = 1.6, 95%CI 1.07–2.34, KM log-rank ChiSq = 11.5, *p* = 0.0007) subgroup, while a statistical trend-level association was observed in the T + subgroup (Cox aHR = 1.5, 95%CI 0.99–2.31, KM log-rank ChiSq = 8.0, *p* = 0.005). No association was observed in the A- and T- MCI subgroups (Table 3, Fig. 2). The analysis was not performed for MCI A-T- and A + T + subgroups, or any of the CU pathology subgroups due to the small number of smokers in each subgroup (ranging between 6 to 16, Table 2).

#### Depression and progression to MCI and/or dementia

A comparison between participants with and without depression in the CU group showed no significant association with progression to MCI or dementia across the A-/A + and T-/T + biomarker subgroups (Table 3, Supplementary eFigure2). Analysis stratified by A-T-/A + T + status was not performed due to the small number of individuals with A + T + pathology and depression (*n* = 12, Table 2). The KM analyses showed no statistically significant difference between CU/Depression risk groups (all *p* values > 0.1).

**Fig. 3:**
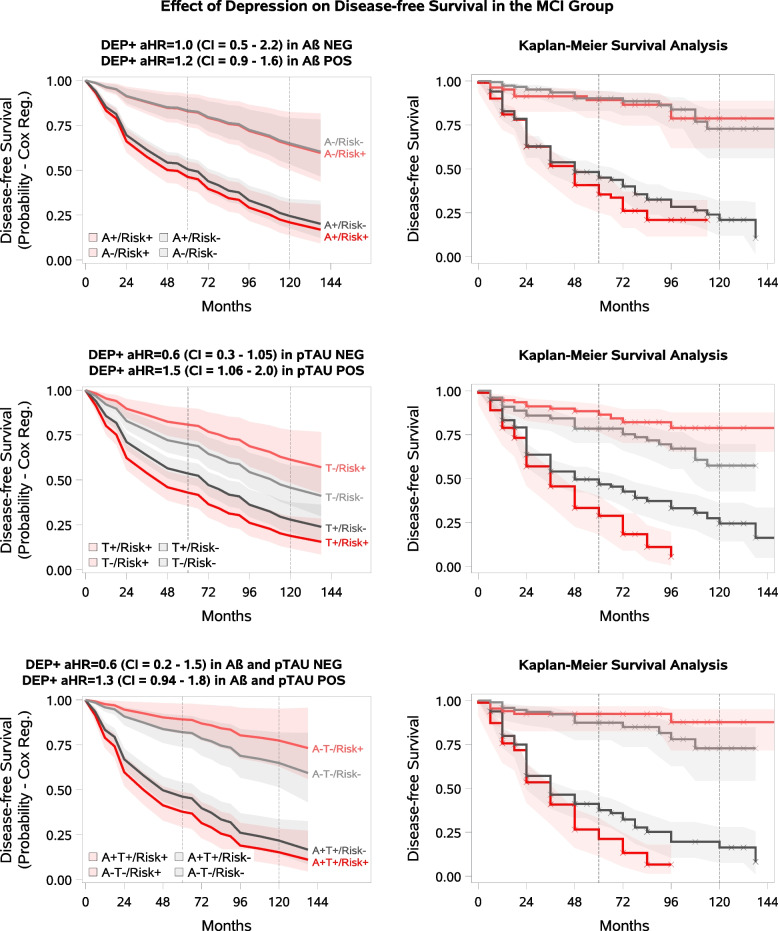
**Depression and Dementia Progression in MCI by beta-amyloid /p-tau Status.** The pale lines in the figure represent the biomarker-negative group, the solid lines represent the biomarker-positive group, the red lines represent the modifiable risk factor-positive group, and the grey lines represent the modifiable risk factor-negative group. The shaded areas represent the confidence intervals. Disease-free survival means no progression to dementia. **A** Depression at baseline as a modifiable risk factor in MCI A-/A + participants. **B** Depression at baseline as a modifiable risk factor in MCI T-/ T + participants. **C** Depression at baseline as a modifiable risk factor in MCI A-T-/A + T + participants

In the MCI group, a significant difference in the risk of progression to dementia was observed between participants with and without depression in the T + subgroup (Cox aHR = 1.5, 95%CI 1.06–2.02, KM log-rank ChiSq = 8.2, *p* = 0.004), and a statistical trend-level associacion was observed in the A + T + subgroup (Cox aHR = 1.3, 95%CI 0.94–1.84, KM log-rank ChiSq = 3.9, *p* = 0.049) (Table 3, Fig. 3). No significant relation was identified between depression and progression to dementia in the biomarker-negative subgroups.

## Discussion

We investigated to what extent the CAIDE dementia risk score, smoking, and depression (history of depression, or current symptoms) as modifiable risk factors were related to clinical progression of cognitive impairment in the presence or absence of Aβ and p-tau pathology. Analyzing the CU and MCI individuals separately, we found that the association of these risk factors with progression varied depending on the presence or absence of AD pathological changes.

The adverse association of the currently studied modifiable risk factors with the occurrence of Aβ and p-tau pathology is well documented in the literature. However, in this study no significant baseline differences were found in the occurrence of AD pathology between the subgroups with and without risk factors such as higher CAIDE score, smoking, or depression. While the influence of these modifiable factors on dementia risk is well established [3, 5, 6, 14, 17, 33, 34], the novelty of our research concerns their role specifically in the presence or absence of AD pathology.

A higher CAIDE score was associated with an increased risk of progression to dementia in MCI participants who were A-, T + , A-T-, and A + T + . Furthermore a statistical trend-level increase of risk was observed in the A + and T- subgroups. Associations were no longer significant when the CAIDE score cut-off was increased to seven, which may be due to smaller size of the higher risk group, since total CAIDE score as a continuous variable was related to an increased progression risk. Since higher CAIDE score was associated with higher progression risk in all almost MCI biomarker subgroups, and results were confirmed by a different unadjusted analytical approach (Kaplan–Meier survival analysis), these findings suggest that addressing modifiable vascular/lifestyle risk factors is critical to reducing the risk of progression due to non-AD pathology. Furthermore, even in the presence of AD pathology, managing these risk factors could significantly reduce the risk of dementia. Recent multimodal prevention models are combining e.g. FINGER lifestyle intervention with putative disease-modifying drugs [56]. The potential added benefit of lifestyle-based interventions would be particularly interesting to investigate in the context of new promising anti-Aβ therapies. Given the higher hazard ratios associated with higher CAIDE score in the non-AD MCI groups, our results further emphasize the importance of managing hypertension, obesity and hyperlipidaemia in dementia prevention, and highlight the potential for dementia risk reduction with vascular/lifestyle-based interventions in a significant group of cognitively impaired people who would most likely not be eligible for e.g. anti-Aβ therapies [57].

The detrimental relationship between depression and dementia is widely supported [6, 9, 33, 34]. Examining history of depression and depressive symptoms together, in the present study an increased risk of cognitive decline related to depression was found in the T + MCI subgroup, with a statistical trend-level association in the A + T + MCI subgroup. No statistically significant association with progression was observed in the A + and biomarker-negative MCI subgroups or in any CU subgroups studied (A + /A-,T + /T-). Notably, there was a significant difference in the prevalence of depression between the CU and MCI groups (17.1% vs 31.4%). One explanation for the link between depression and cognitive decline could be the serotonin and cholinergic deficits described as a consequence of depression [53, 58–62]. Depression is also associated with other risk factors for dementia, such as reduced physical activity, sleep disturbances, altered diet, and increased smoking [5, 63, 64]. Therefore, both direct and indirect effects of depression may increase the risk of dementia. An ongoing debate exists regarding whether mid- and late-life depression should be interpreted as a prodrome of dementia or as an independent risk factor [65, 66]. Our results highlight the importance of paying special attention to depressive symptoms, even in the presence of AD pathology, irrespective of whether depression is a risk factor or a consequence of the disease.

There is a well-established link between social activity and lower levels of depression [67, 68]. Social connections—including those facilitated by social media—have become increasingly important. Particularly for older adults who are at risk of isolation, social media platforms offer opportunities to maintain and enhance social interactions [69, 70]. Research suggests that certain types of social media use can have a positive impact on mental health, which may help to reduce certain dementia risk factors [71, 72]. Including social media use in lifestyle interventions may improve mental health and reduce the risk of dementia. Future research should explore the benefits of social media in vulnerable populations.

There was a significant association between smoking and progression to dementia in the MCI A + subgroup, and a trend-level association in the MCI T + subgroup, while the MCI A- and T- subgroups showed no correlation. Several mechanisms may explain the association between smoking and dementia [14, 30–32]. Some studies suggested that smoking may directly affect Aβ-associated degeneration [11, 14, 32, 73], accelerating its onset. In addition, smoking is known to have adverse effects on the vasculature [14, 30–32]. Other studies have shown that any factor that reduces oxygen supply leads to local Aβ deposition [74–76]. Preclinical research using AD-induced hypoxic models confirms that reduced brain vascularisation caused by smoking may contribute to an increased risk of dementia [74]. It should also be considered that smokers' lifestyles are often associated with other risk factors, such as a sedentary lifestyle or poor diet [77].

When interpreting our results for p-tau, it is important to note that the tau classification was based on CSF p-tau181, which is included in the Alzheimer's Association Workgroup Recommendation 2024 as a Core1 T_1_ biomarker and is recommended to be used primarily in conjunction with CSF Aβ42, as it has greater diagnostic value in this context. In addition, CSF p-tau181 becomes abnormal at the same time as amyloid PET and before tau PET. It is thought that the secretion of these tau fragments may represent a physiological response to Aβ plaques and may link Aβ proteinopathy to early tau proteinopathy [54]. At the same time, it is worth highlighting the role of p-tau181 as a prognostic factor. In our previous meta-analysis based on several studies measuring CSF p-tau181, we found that individuals identified as A + T + (using CSF p-tau181) had significantly higher odds ratios for cognitive decline compared to the A + or A + T- groups [41].

Finally, it is important to note that no significant association was identified between progression and the risk factors tested (CAIDE score, depression) in any of the CU biomarker subgroups. Given the well-established deleterious role of these risk factors in cognitive decline, we have two possible explanations. Firstly, the relatively low progression rate in the CU group (19.1% compared with 36.2% in MCI) may have reduced the statistical power to detect significant associations. Secondly, the median follow-up of the healthy group was four years, which may be insufficient for the adverse effects of these risk factors to become apparent in individuals who are cognitively intact.

### Strenghts and limitations

This study used a large, well-characterised sample from the ADNI, including 434 CU and 611 MCI individuals, with a median follow-up of four years. However, the present study has several limitations. Aβ status was determined based on PET scans in most participants, and on CSF in the rest. Although PET is known to be more sensitive, both methods are widely used in practice, the concordance between the two methods is high, and CSF measurement is more widely available for financial reasons [38, 78].

The CAIDE scoring system provides a comprehensive and easy-to-use overview of cardiovascular and lifestyle risk factors. However, CAIDE was initially developed for a middle-aged population, and in the original study, it was used to predict the risk of dementia over 20 years. Since then, there have been examples of its use with shorter follow-ups and in older patients [3]. There is no uniform recommendation for the point value to separate the high- and low-risk groups so that this cut-off may differ in other populations. Nevertheless, utilizing the median for separating groups is appropriate for identifying the risk due to CAIDE factors. It should be noted that the lack of data on physical activity may lead to an underestimation of the association. However, the effect of physical activity is less weighted, changing the CAIDE score by only one point, compared with other modifiable risk factors, each of which contributes two points. Importantly, the accuracy and validity of cognitive tests and the CAIDE score may be influenced by cultural differences [79, 80]. To ensure that these assessments are globally applicable, future research should focus on validating and modifying them for a range of populations.

In the case of depression, it should be noted that the participants were classified based on their medical history. The severity of the depression or whether it was a late or early onset could not be considered. A more accurate classification method could further refine the results. Symptoms of depression at baseline were assessed by a detailed and comprehensive neuropsychiatric inventory developed for the detection of behavioral disturbances in dementia [43]. However, it has been utilised in preceding clinical trials with participants with MCI and CU and has been demonstrated to be a valid and reliable measure [46–48, 81]. Nevertheless, a clinically structured interview was not performed to diagnose depressive disorders according to the Diagnostic Diagnostic and Statistical Manual of Mental Disorders (DMS) [82]. In terms of smoking habits, only self-reported information was utilized, and a limitation of the study is the lack of consideration of the severity of smoking. Another limitation is that the potential confounding effect of these risk factors on each other is not included in our calculations. It also should be noted that the ADNI cohort is skewed towards white individuals and those with higher levels of education. This latter fact may restrict the generalisability of the findings to a more diverse population.

Another limitation is that the analyses could not take into account the effects of medications used for depression, hyperlipidaemia and hypertension. Therefore, these conditions were only included as categorical variables, as we could not take into account their treated or untreated status.

A limitation of the observations for CU participants is that analyses for smoking could not be performed due to the small number of cases, and analyses for depression were only partially performed. Additionally, the results for CAIDE scores and depression in the CU group are based on a moderately small sample size, resulting in lower statistical power compared to the MCI group.

We emphasise that our study aimed to investigate the role of modifiable risk factors in different biomarker subgroups, not to compare their effect between these different biomarker states. Due to statistical power limitations for interaction analyses, it remains unclear if the associations of CAIDE score, smoking and depression with clinical progression differ between the different biomarker subgroups.

## Conclusion

Even after the onset of AD pathology, addressing modifiable risk factors remains critical to reducing the risk of dementia. As the effects of vascular/lifestyle-based interventions on dementia risk reduction are currently being investigated in randomized controlled trials, a key focus for future studies should be how the presence or absence of AD pathology may impact intervention effects, and potential added benefit of combining lifestyle-based and pharmacological therapies in populations who already have cognitive impairment and AD pathology.

## Supplementary Information

Supplementary Material 1.

## Data Availability

No datasets were generated or analysed during the current study.

## Abbreviations

A-: Non-pathologic levels of beta-amyloid
A +: Pathologic levels of beta-amyloid
Aβ: Beta-amyloid
AD: Alzheimer’s disease
ADNI: Alzheimer’s Disease Neuroimaging Initiative
aHR: Adjusted Hazard Ratio
ChiSq: Chi-square
CI: Confidance interval
Cox: Cox regression analyses
CU: Cognitively unimpaired
CSF: Cerebrospinal fluid
HR: Hazard ratio
KM: Kaplan–Meier analyses
MCI: Mild cognitive impairment
PET: Positron emission tomography
p-tau: Phosphorylated tau
T-: Non-pathologic levels of phosphorylated tau
T +: Pathologic levels of phosphorylated tau
