# Supplementary material for: Association of modifiable risk factors with progression to dementia in relation to amyloid and tau pathology
Source: Alzheimers Res Ther. 2024 Oct 26;16:238. doi: 10.1186/s13195-024-01602-9 (PMC11515263; doi:10.1186/s13195-024-01602-9)
Supplement: Supplementary file 1 — Supplementary Material 1. [file 13195_2024_1602_MOESM1_ESM.docx]

**Supplementary material**

**Zsolt Huszár, Gábor Csukly et al.: Association of modifiable risk factors with progression to dementia in relation to amyloid and tau pathology**

**Table of contents**

**Appendix 1:** ADNI - Alzheimer's Disease Neuroimaging Initiative

**eTable 1:** CAIDE score system

**eFigure 1**: CAIDE score and Dementia Progression in CU by beta-amyloid /p-tau Status

**eFigure 2**: Depression and Dementia Progression in CU by beta-amyloid /p-tau Status

**Appendix 1** ADNI - Alzheimer's Disease Neuroimaging Initiative

Data used in the preparation of this article were obtained from the Alzheimer’s Disease Neuroimaging Initiative (ADNI) database (adni.loni.usc.edu). The ADNI was launched in 2003 as a public-private partnership, led by Principal Investigator Michael W. Weiner, MD. The primary goal of ADNI has been to test whether serial magnetic resonance imaging (MRI), positron emission tomography (PET), other biological markers, and clinical and neuropsychological assessment can be combined to measure the progression of mild cognitive impairment (MCI) and early Alzheimer’s disease (AD). For up-to-date information, see www.adni-info.org.

**Acknowledgement**:

Data collection and sharing for this project was funded by the Alzheimer's Disease Neuroimaging Initiative (ADNI) (National Institutes of Health Grant U01 AG024904) and DOD ADNI (Department of Defense award number W81XWH-12-2-0012). ADNI is funded by the National Institute on Aging, the National Institute of Biomedical Imaging and Bioengineering, and through generous contributions from the following: AbbVie, Alzheimer’s Association; Alzheimer’s Drug Discovery Foundation; Araclon Biotech; BioClinica, Inc.; Biogen; Bristol-Myers Squibb Company; CereSpir, Inc.; Cogstate; Eisai Inc.; Elan Pharmaceuticals, Inc.; Eli Lilly and Company; EuroImmun; F. Hoffmann-La Roche Ltd and its affiliated company Genentech, Inc.; Fujirebio; GE Healthcare; IXICO Ltd.; Janssen Alzheimer Immunotherapy Research & Development, LLC.; Johnson & Johnson Pharmaceutical Research & Development LLC.; Lumosity; Lundbeck; Merck & Co., Inc.; Meso Scale Diagnostics, LLC.; NeuroRx Research; Neurotrack Technologies; Novartis Pharmaceuticals Corporation; Pfizer Inc.; Piramal Imaging; Servier; Takeda Pharmaceutical Company; and Transition Therapeutics. The Canadian Institutes of Health Research is providing funds to support ADNI clinical sites in Canada. Private sector contributions are facilitated by the Foundation for the National Institutes of Health (www.fnih.org). The grantee organization is the Northern California Institute for Research and Education, and the study is coordinated by the Alzheimer’s Therapeutic Research Institute at the University of Southern California. ADNI data are disseminated by the Laboratory for Neuro Imaging at the University of Southern California.

**ADNI sponsors**:

Data collection and sharing for this project was funded by the Alzheimer's Disease Neuroimaging Initiative (ADNI) (National Institutes of Health Grant U01 AG024904) and DOD ADNI (Department of Defense award number W81XWH-12-2-0012). ADNI is funded by the National Institute on Aging, the National Institute of Biomedical Imaging and Bioengineering, and through generous contributions from the following: AbbVie, Alzheimer’s Association; Alzheimer’s Drug Discovery Foundation; Araclon Biotech; BioClinica, Inc.; Biogen; Bristol-Myers Squibb Company; CereSpir, Inc.; Cogstate; Eisai Inc.; Elan Pharmaceuticals, Inc.; Eli Lilly and Company; EuroImmun; F. Hoffmann-La Roche Ltd and its affiliated company Genentech, Inc.; Fujirebio; GE Healthcare; IXICO Ltd.; Janssen Alzheimer Immunotherapy Research & Development, LLC.; Johnson & Johnson Pharmaceutical Research & Development LLC.; Lumosity; Lundbeck; Merck & Co., Inc.; Meso Scale Diagnostics, LLC.; NeuroRx Research; Neurotrack Technologies; Novartis Pharmaceuticals Corporation; Pfizer Inc.; Piramal Imaging; Servier; Takeda Pharmaceutical Company; and Transition Therapeutics. The Canadian Institutes of Health Research is providing funds to support ADNI clinical sites in Canada. Private sector contributions are facilitated by the Foundation for the National Institutes of Health (www.fnih.org). The grantee organization is the Northern California Institute for Research and Education, and the study is coordinated by the Alzheimer’s Therapeutic Research Institute at the University of Southern California. ADNI data are disseminated by the Laboratory for Neuro Imaging at the University of Southern California.

**Methods used in patient level data analyses in the ADNI database**

We used patient level data from the ADNI database to calculate HRs and ORs. We used the 18F-Florbetapir (AV45) PET data as default Amyloid measurement, where it was available. Florbetapir standardized uptake value ratio (SUVR) was created by averaging the four cortical regions and dividing it by the cerebellum as reference. According to the ADNI recommendation we applied the SUVR cutoff of 1.11 and used the whole cerebellum region as reference(1) . In a previous study Clark et. al. (2012)(2) showed that Florbetapir positivity using the same cutoff strongly correlated with post-mortem autopsy results. If PET data was not available, we used Amyloid 1-42 CSF measurements to maximize the size of the analysis sample. According to Hansson et al. (2018)(3) we applied a cutoff of 977 pg/ml for Amyloid 1-42 measurements (Roche Elecsys Abeta42 CSF measures were used), since this cutoff value showed the highest agreement with Amyloid PET results (overall percent agreement was 87% (95%CI = 84.2-89.5%)). Subjects were defined as p-tau positive by CSF p-tau levels (INNO-BIA AlzBio3 CSF measures were used) were up to 23 pg/ml, since Shaw et al (2009)(4) showed on autopsy-based Alzheimer cases that this cutoff has the best classification power.

Hazard Ratio (HR) calculation in the ADNI database

In the ADNI database HRs were calculated from a Cox Proportional Hazard Model (PROC PHREG in SAS 9.4). Conversion to MCI or conversion to dementia were the dependent (predicted) variables in separate models, while Amyloid positivity applying the above-mentioned procedures and cutoffs served as predictor variables (unadjusted analyses). In case of the adjusted analyses, we also included age, gender, education, baseline MMSE score and APOE status as covariates.

**Supplementary table 1** CAIDE score system

| **Risk factors** | **Points** |
| --- | --- |
| Age: < 47 years  47 – 53 years  > 53 years | 0  3  4 |
| Education: ≥ 10 years  7-9 years  < 7 years | 0  2  3 |
| Gender: Female  Male | 0  1 |
| Blood pressure: ≤140 mm hg  >140 mm hg | 0  2 |
| Body-mass index: ≤ 30kg/m^2^  > 30kg/m^2^ | 0  2 |
| Total cholesterol: ≤ 6.5 mmol/L  > 6.5 mmol/L | 0  2 |
| Physical activity^*^: yes  no | 0  1 |

* not available in ADNI.

**
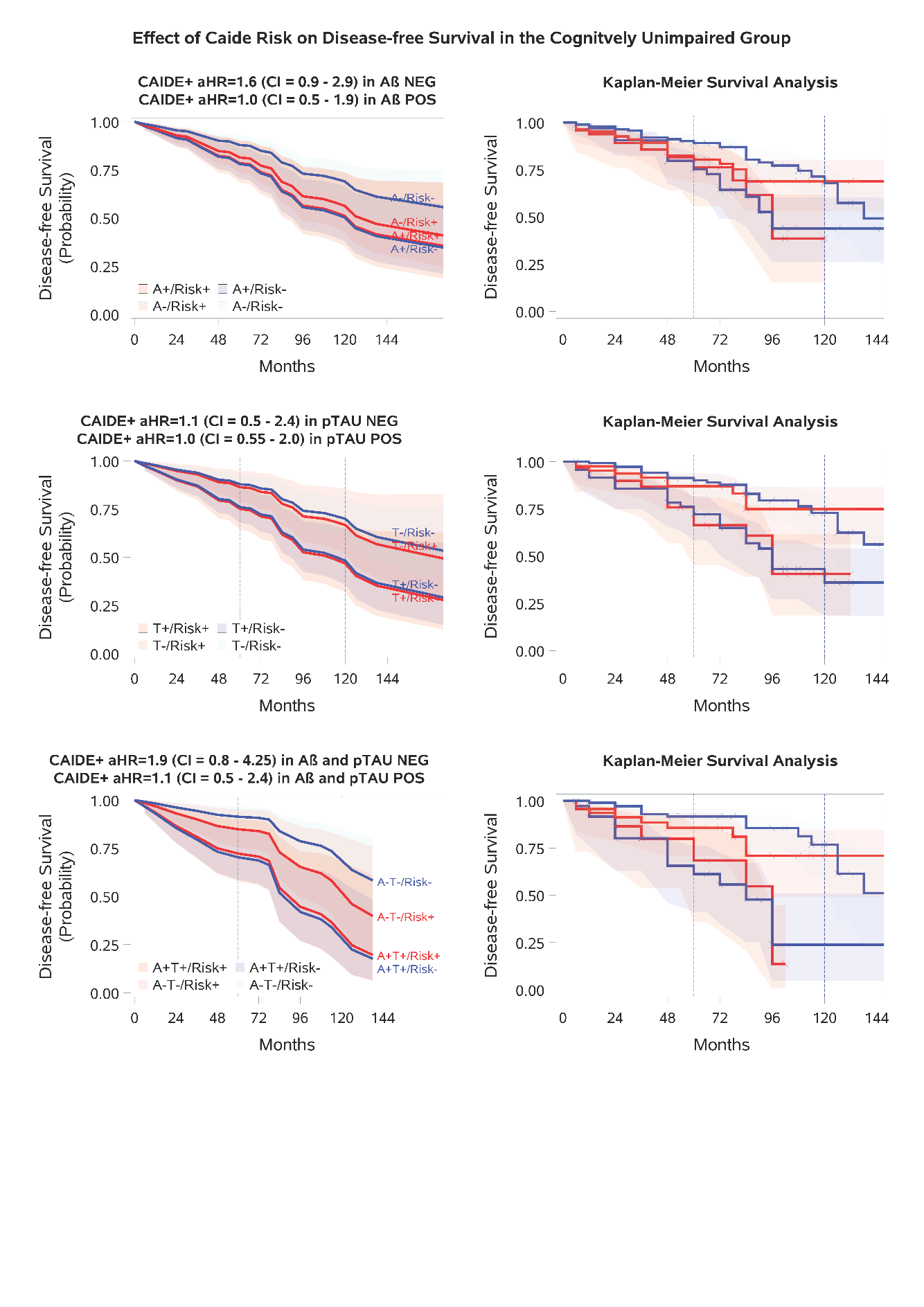
eFigure 1**: CAIDE score and Dementia (MCI) Progression in CU by beta-amyloid /p-tau Status

The continuous lines represent the biomarker-positive groups, the dotted lines the biomarker-negative groups, the red lines the modifiable risk factor-positive, and the blue/grey lines the modifiable risk factor-negative groups. The shaded areas represent the confidence interval. Disease-free survival means not converted to dementia.

A. CAIDE at baseline as a modifiable risk factor in CU A-/A+ participants.

B. CAIDE at baseline as a modifiable risk factor in CU T-/ T+ participants.

C. CAIDE at baseline as a modifiable risk factor in CU A-T-/A+T+ participants.

**eFigure 2**: Depression and Dementia (MCI) Progression in CU by beta-amyloid /p-tau Status

**
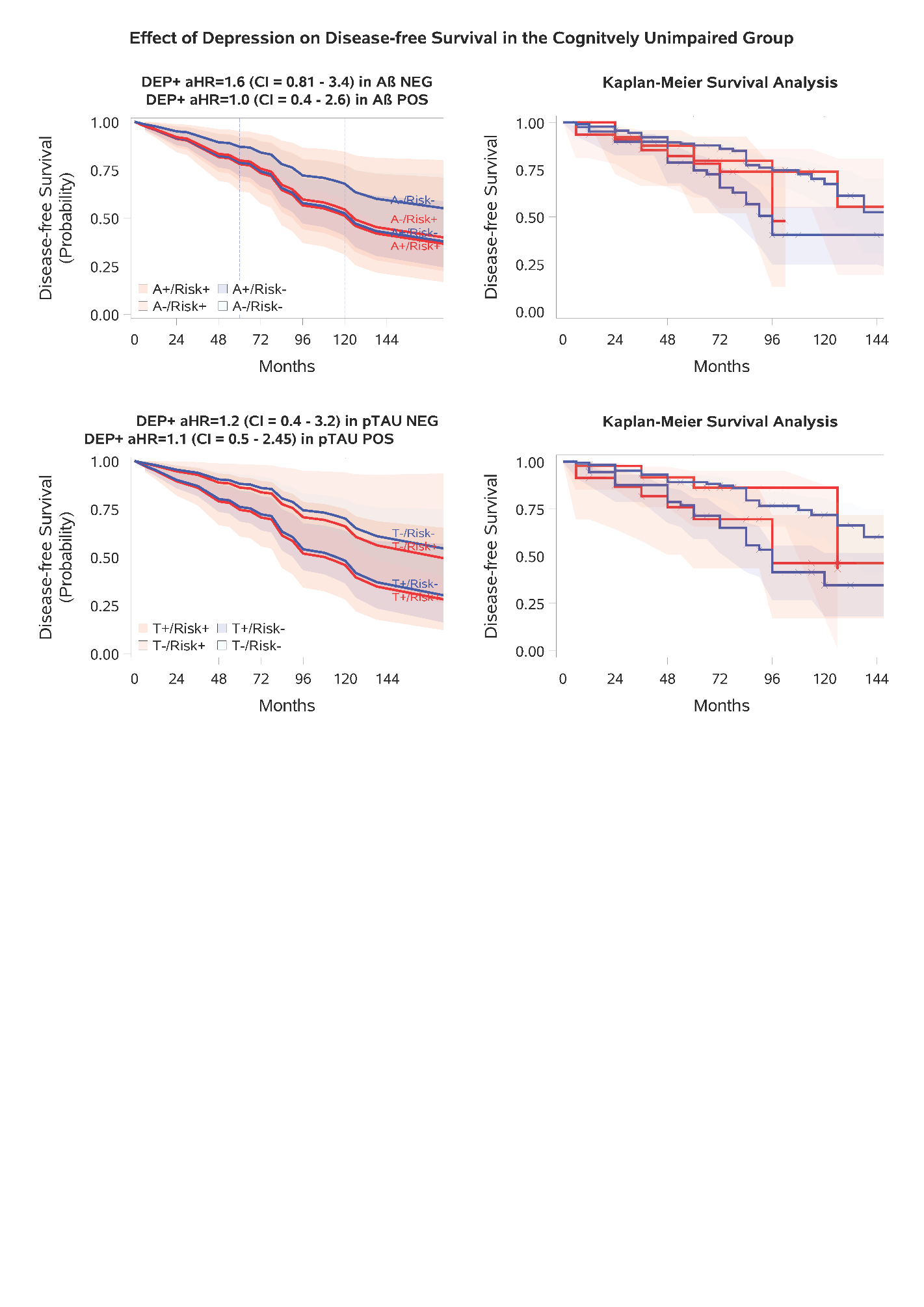
**

The continuous lines represent the biomarker-positive groups, the dotted lines the biomarker-negative groups, the red lines the modifiable risk factor-positive, and the blue/grey lines the modifiable risk factor-negative groups. The shaded areas represent the confidence interval. Disease-free survival means not converted to dementia.

A. Depression at baseline as a modifiable risk factor in CU A-/A+ participants.

B. Depression at baseline as a modifiable risk factor in CU T-/ T+ participants.

C. Depression at baseline as a modifiable risk factor in CU A-T-/A+T+ participants.

**References**

1. Landau S, William J. Florbetapir processing methods. In: Helen Wills Neuroscience Institute UBaLBNL, editor. 2015.

2. Clark CM, Schneider JA, Bedell BJ, Beach TG, Bilker WB, Mintun MA, et al. Use of florbetapir-PET for imaging beta-amyloid pathology. Jama. 2011;305(3):275-83.

3. Hansson O, Seibyl J, Stomrud E, Zetterberg H, Trojanowski JQ, Bittner T, et al. CSF biomarkers of Alzheimer's disease concord with amyloid-β PET and predict clinical progression: A study of fully automated immunoassays in BioFINDER and ADNI cohorts. Alzheimers Dement. 2018;14(11):1470-81.

4. Shaw LM, Vanderstichele H, Knapik-Czajka M, Clark CM, Aisen PS, Petersen RC, et al. Cerebrospinal fluid biomarker signature in Alzheimer's disease neuroimaging initiative subjects. Ann Neurol. 2009;65(4):403-13.
